# Irrational beliefs in Bahasa Malaysia and Mandarin speaking populations: the cross-cultural validation of the irrational performance beliefs inventory

**DOI:** 10.1186/s40359-025-03579-y

**Published:** 2025-12-01

**Authors:** Alena Michel-Kröhler, Michael Wong, Martin J. Turner

**Affiliations:** 1 Department of Clinical Psychology and Neuropsychology, Institute for Psychology, Johannes Gutenberg-University Mainz, Mainz, Germany; 2 Department of Neuropsychology and Psychological Resilience Research, Central Institute of Mental Health, Mannheim, Germany; 3 Department of Sport and Exercise, Staffordshire University, Stoke-On-Trent, UK; 4 Department of Psychology, Institute of Sport, Manchester Metropolitan University, Manchester, UK

**Keywords:** Psychometrics, REBT, Thai-iPBI, IPBI Korean version, CBT

## Abstract

**Background:**

Irrational beliefs as conceptualized within Rational Emotive Behavior Therapy (REBT) are maladaptive ideas about the world that perpetuate ill mental health and psychological distress. However, our understanding of the role of irrational beliefs in psychopathology is heavily reliant on the data from Western samples, chiefly the United Kingdom and the United States. Thus, to help bridge the gap between East and West, we must first understand the extent to which our measures are suitable for cross-cultural purposes. In this article we present the translation and validity testing of two versions of the irrational Performance Beliefs Inventory (iPBI) – a Mandarin language version and a Bahasa Malaysia language version.

**Methods:**

For this purpose, 239 Bahasa Malaysia speaking participants and 180 Mandarin speaking participants (f = 139, m = 280, *M*_age_ = 23.05, *SD*_age_ = 6.02, age range: 16–50 years) took part in the study. After a detailed translation process, we carried out confirmatory factor analyses to test whether the expected four-factor-structure can be confirmed in these Bahasa Malaysia and Mandarin speaking subsamples.

**Results:**

Results showed a partially inadequate model fit (Bahasa Malaysia version: CFI = .841, RMSEA = .072; Mandarin version: CFI = .866, RMSEA = .066). In addition, we conducted exploratory analyses and applied existing versions of the iPBI, namely the existing Thai and Korean versions, to our sample to examine whether they provide a better model fit. In addition, construct validity (i.e., convergent, divergent, and concurrent validity) indicated partial validity, with mixed relationships between irrational beliefs and trait anxiety, anger, and depression for the different iPBI versions.

**Conclusion:**

The current findings indicate that further psychometric work is necessary before Bahasa Malaysia and Mandarin versions of the iPBI can be adequately applied in research and practice. We discuss possible shortcomings and provide suggestions for further research.

**Supplementary Information:**

The online version contains supplementary material available at 10.1186/s40359-025-03579-y.

## Background

For many people, performance is an important and unavoidable part of daily life. This could be performance in private life (e.g., being a good mother/father/friend), at school or university (e.g., getting good grades/giving good presentations), at work (e.g., closing the most deals/winning the most customers) or in sport (e.g., achieving the best performance, being better than the opponent). Indeed, performance in such areas can help shape the life course of individuals and organizations. Performance is ubiquitous in our lives, so to speak, which can lead people to place an exaggerated personal importance on performance behaviors and outcomes [20]. Furthermore, performance is not always automatically associated with success. There may be repeated experiences of failure, injustice, rejection, or similar challenging events to which a person may respond with healthy or unhealthy behaviors [9]. One approach to understanding human goal striving posits that rigid, extreme, and irrational beliefs about performance-related factors can paradoxically impede progress toward desired outcomes. This approach is consistent with Rational Emotive Behavior Therapy (REBT, [19]), which emphasizes the crucial role of beliefs in the pursuit of goals and the development of psychopathology when goals are impeded. In REBT, rational and irrational beliefs (B) are at the center of a transactional GABC emotion reactivity process whereby adverse (A) occurrences to one’s goals (G) bring about negative emotional and behavioral consequences (C). Irrational beliefs in response to, or on approach to, adversity underpin unhealthy consequences, and rational beliefs underpin healthy consequences [18, 49, 56]. Irrational beliefs are illogical, unpragmatic, and unempirical, and in contrast, rational beliefs are logical, pragmatic, and empirical. Irrational beliefs are at the heart of treatment in (specific) REBT [56]. The application of the GABCDE framework within REBT (outlined first by Ellis [18], and elaborated by Turner [56]) involves a targeted intervention. Initially, this involves a thorough assessment of deeply held irrational beliefs and recognizing their role in maladaptive emotional and behavioral outcomes, rather than attributing these outcomes solely to the eliciting event. Subsequently, these beliefs are rigorously challenged and weakened, following which rational alternatives are developed and strengthened to improve the individual’s psychological well-being. However, research investigating the use of REBT in performance situations has so far mainly been conducted in Western, particularly European, countries (for two exceptions in sport samples see [13, 45]). Consequently, it is not clear whether and to what extent REBT can be applied in Eastern samples. Therefore, the priority for the application of REBT in Eastern populations should be to develop a linguistically and psychometrically valid measure of irrational beliefs as a function of the language spoken in the respective region to help researchers and practitioners identify individuals vulnerable to irrational beliefs, practice REBT, and finally test the effects of REBT on irrational beliefs (cf. [9]).

Turner and colleagues [53] developed the irrational Performance Beliefs Inventory (iPBI) as the first measure of irrational beliefs in performance areas such as sport, education, business, and the military. The iPBI consists of 28 items that capture irrational performance beliefs according to four core dimensions of REBT theory [4, 17, 19]. The first core belief, Demandingness, also known as Primary Irrational Belief (PIB) can be defined as rigid assertion of demands that certain desired conditions must or must not exist [15]. Three other core beliefs are subordinate to PIBs: (a) Awfulizing (AWF) as the extreme overestimating the consequences of past, present, and future events while being unable to recognize that there could be worse events [12], (b) Low-frustration tolerance (LFT) as the belief that when people do not get what they think they should get, they conclude that the situation is intolerable, and they cannot bear it [16], and (c) Depreciation (DEP) as the tendency to make global judgements (i.e., overgeneralizations) about oneself, others, and the world [12]. Previous studies confirmed the construct, concurrent, and predictive validity of the iPBI [54, 55], as well as its test–retest reliability [54, 55].

To assess irrational performance beliefs across different cultures, various translations of the iPBI have been developed and subjected to validity testing: Korean [8], German [38], Hungarian [52], Persian [39], Thai [9], and Turkish [57]. In the Thai, Korean, Persian, and German translation, which are based on the original 28-item iPBI version, the measure has proven to be internally consistent (α > 0.69). Further construct validation was confirmed across cultures by low to moderate correlations with measures of anxiety and depression. However, the 28-item version was not fully replicated in any of the mentioned translations, resulting in versions of varying lengths with 26 items [38], 22 items [39] or 20 items [8, 9]. At present, however, it is not known to what extent irrational beliefs are also relevant and transferable to other regions of Asia such as Malaysia and other languages such as Bahasa Malaysia or Mandarin.

From a cultural perspective, adapting to these two languages will allow access to a large part of the Malaysian community, as Bahasa Malaysia is the official language and Mandarin is becoming increasingly important due to its increased social status within Malaysia’s Chinese community, historically differentiated by dialect [1, 22, 41]. While the Malay culture is diverse and dynamic, with Islam playing a significant role in shaping its values and norms, the Chinese community maintains a strong cultural identity rooted in Confucian principles and a focus on family values. This leads to subtle differences in their fundamental attitudes and practices, particularly in the business environment. Although both communities maintain their distinct identities, there is also a shared understanding of Malaysian culture and a general spirit of coexistence. For example, both communities value maintaining harmony, avoiding conflict in communication, and respecting elders [22, 59]. From a practical perspective, validating questionnaires in different languages increases accessibility, enables diverse populations to participate in research and clinical practice, and supports more inclusive health and psychological interventions [11]. From a researcher’s perspective, this is particularly relevant in relation to improving the international comparability of research results, enabling large-scale intercultural studies, and improving the generalizability of findings [2].

Therefore, the aim of the study was therefore the development and preliminary validation of two new Asian language versions of the irrational Performance Beliefs Inventory –a Bahasa Malaysia and a Mandarin version (two prominent languages spoken in Malaysia), which can be applied mainly in Malaysia but also in other regions of Asia. To achieve this aim, the original iPBI was translated from English into Bahasa Malaysia (*hereinafter referred to as* iPBI-Malay) and Mandarin (*hereinafter referred to as* iPBI-Mandarin). Using the translated versions, we assessed construct validity in terms of factorial validity using confirmatory factor analyses (CFAs) and determined convergent and divergent validity using the different subscales of the Shortened General Attitude and Belief Scale (SGABS; [34]). In addition, we calculated the correlations between the new measures and the State-Trait Personality Inventory (STPI; [46]) to check concurrent validity, determined differences between participants’ sex and calculated correlations with participants’ age.

Finally, since two versions of the iPBI have already been validated in Eastern working populations, namely a Thai and a Korean version, we exploratively checked the model fit of these versions for our data. In addition, we repeated the already mentioned analyses to determine the convergent, divergent, and concurrent validity for these two versions to identify the most appropriate measurement model for the Mandarin and Bahasa Malay speaking populations.

## Method & materials

### Participants

For conducting confirmatory factor analysis (CFA), previous iPBI research has used a participant:item ratio of 5:1 [9], but guidelines indicate an a priori participant:item ratio of 10:1 as more suitable (e.g., [5, 10]). Thus, we aimed to recruit between *n* = 140 and *n* = 280 participants for both Mandarin and Bahasa Malaysia versions of the iPBI to complete the 28-item iPBI. Overall, we recruited 422 participants from 13 Malaysian states and 3 federal territories. We removed three participants from the analyses because their age deviated significantly from the mean distribution of the sample (≥ 88 years). Thus, the final sample consists of 419 participants (f = 139, m = 280, *M*_age_ = 23.05, *SD*_age_ = 6.02, age range: 16–50 years), which can be divided in two subsamples for data analyses:

#### Subsample 1 – Mandarin speaking sample

One hundred eighty participants (f = 62, m = 118, *M*_age_ = 21.26, *SD*_age_ = 5.68, age range: 16–48 years). Participants of Subsample 2 had mainly the second highest education level (i.e., 7–11 years [O Level], or 12–13 years [A Level], *n* = 94), were mostly Buddhist (*n* = 118) and belonged mainly to the Chinese community (*n* = 162; for more details see Supplement Table A1).

#### Subsample 2 – Bahasa Malaysia speaking sample

Subsample 2 consisted of 239 participants (f = 77, m = 162, *M*_age_ = 24.41, *SD*_age_ = 5.94, age range: 16–50 years). Most of them had the third highest education level (i.e., 1–4 years diploma or degree at a college or university, *n* = 132), were mostly Muslim (*n* = 220) and belonged mainly to the Malay community (*n* = 196; for more details see Supplement Table A1).

### Measures

For the present study, we translated the original English versions of the irrational Performance Beliefs Inventory (iPBI), the Shortened General Attitude and Belief Scale (SGABS) and the State-Trait Personality Inventory (STPI) into two languages, Mandarin and Bahasa Malaysia. Descriptive statistics and reliability coefficients (Cronbach’s alpha; α) for the Mandarin speaking sample are reported in the first third of Table 1 and for the Bahasa Malaysia speaking sample in the first third of Table 3. For the translation process, we used a modified team approach (see [6, 14]), which includes five successive steps: translation, review, adjudication, pre-testing and documentation (short: TRAPD; 2003, see also [48]). The TRAPD approach was chosen because research has shown that a committee or team approaches produce the most effective translations compared with other approaches [23, 25]. In more detail, five translators (three Bahasa Malaysia and two Mandarin translators) produce initial translations independent of each other. An expert panel reviewed the translations together with the translators and the second author. After that, a pre-test translation was produced.

**Table 1:** Descriptive statistics, inter-correlations for the iPBI dimensions, and correlation coefficients for the Mandarin speaking sample

	***M* (*SD*)**	**α**	**PIB**	**LFT**	**AWF**	**DEP**	**iPBI Composite score**
iPBI-Mandarin							
Primary irrational beliefs (PIB)	3.51 (0.46)	.62	-				
Low frustration tolerance (LFT)	3.49 (0.51)	.66	.43^***^	-			
Awfulization (AWF)	3.40 (0.48)	.75	.72^***^	.56^***^	-		
Depreciation	2.81 (0.71)	.83	.38^***^	.52^***^	.59^***^	-	
iPBI Composite score	3.25 (0.46)	.89	-	-	-	-	-
* SGABS*							
Need for achievement	3.34 (0.78)	.76	.29^***^	.56^***^	.49^***^	.55^***^	.60^***^
Need for approval	2.91 (0.82)	.71	.48^***^	.37^***^	.51^***^	.31^***^	.49^***^
Need for comfort	3.09 (0.75)	.76	.23^***^	.30^***^	.33^***^	.38^***^	.41^***^
Demand on fairness	3.62 (0.68)	.75	.51^***^	.36^***^	.53^***^	.27^***^	.48^***^
Self-downing	2.52 (0.95)	.89	.30^***^	.46^***^	.49^***^	.68^***^	.65^***^
Other downing	3.00 (0.74)	.61	.37^***^	.31^***^	.40^***^	.31^***^	.42^***^
Total irrationality	3.09 (0.56)	.90	.49^***^	.57^***^	.64^***^	.62^***^	.73^***^
* STPI*							
Anxiety	22.14 (3.43)	.76	.16	.20	.25^**^	.27^**^	.29^**^
Anger	16.39 (6.26)	.87	.05	.30^***^	.19	.35^***^	.31^***^
Depression	24.26 (2.73)	.79	<.01	.16	.14	.16	.16
Thai version							
Primary irrational beliefs (PIB)	3.52 (0.42)	.51	-				
Low frustration tolerance (LFT)	3.49 (0.51)	.65	.40^***^	-			
Awfulization (AWF)	3.46 (0.48)	.75	.63^***^	.57^***^	-		
Depreciation	2.83 (0.72)	.83	.33^***^	.50^***^	.52^***^		
iPBI Composite score	3.29 (0.44)	.88	-	-	-	-	-
* SGABS*							
Need for achievement	3.34 (0.78)	.76	.25^**^	.55^***^	.43^***^	.54^***^	.59^***^
Need for approval	2.91 (0.82)	.71	.45^***^	.40^***^	.49^***^	.31^***^	.49^***^
Need for comfort	3.09 (0.75)	.76	.25^**^	.34^***^	.30^***^	.35^***^	.40^***^
Demand on fairness	3.62 (0.68)	.75	.48^***^	.36^***^	.51^***^	.27^***^	.48^***^
Self-downing	2.52 (0.95)	.89	.25^**^	.47^***^	.44^***^	.67^***^	.63^***^
Other downing	3.00 (0.74)	.61	.36^***^	.32^***^	.40^***^	.31^***^	.43^***^
Total irrationality	3.09 (0.56)	.90	.46^***^	.58^***^	.60^***^	.60^***^	.72^***^
* STPI*							
Anxiety	22.14 (3.43)	.76	.20	.21^*^	.24^*^	.27^**^	.30^***^
Anger	16.39 (6.26)	.87	.09	.31^***^	.17	.34^***^	.31^***^
Depression	24.26 (2.73)	.79	.01	.15	.13	.18	.17
Korean version							
Primary irrational beliefs (PIB)	3.65 (0.43)	-	-				
Low frustration tolerance (LFT)	3.60 (0.38)	.57	.38^***^	-			
Awfulization (AWF)	3.40 (0.48)	.75	.63^***^	.59^***^	-		
Depreciation (DEP)	2.83 (0.72)	.83	.29^***^	.52^***^	.59^***^	-	-
iPBIK Composite score	3.30 (0.43)	.88	-	-	-	-	-
* SGABS*							
Need for achievement	3.34 (0.78)	.76	.26^**^	.56^***^	.49^***^	.54^***^	.61^***^
Need for approval	2.91 (0.82)	.71	.34^***^	.39^***^	.51^***^	.31^***^	.47^***^
Need for comfort	3.09 (0.75)	.76	.14	.33^***^	.32^***^	.35^***^	.39^***^
Demand on fairness	3.62 (0.68)	.75	.49^***^	.36^***^	.52^***^	.27^***^	.47^***^
Self-downing	2.52 (0.95)	.89	.20^*^	.48^***^	.49^***^	.67^***^	.65^***^
Other downing	3.00 (0.74)	.61	.31^***^	.31^***^	.39^***^	.31^***^	.41^***^
Total irrationality	3.09 (0.56)	.90	.39^***^	.58^***^	.64^***^	.60^***^	.72^***^
* STPI*							
Anxiety	22.14 (3.43)	.76	.09	.23^*^	.25^**^	.27^**^	.29^**^
Anger	16.39 (6.26)	.87	<.01	.30^***^	.19	.34^***^	.31^***^
Depression	24.26 (2.73)	.79	.04	.18	.14	.18	.19

*p*-values were corrected for multiple comparisons using Holm’s method

*M* Mean, *SD* Standard deviation, *α* Cronbach’s alpha, *iPBI* irrational Performance Beliefs Inventory, *SGABS* Shortened General Attitude and Belief Scale, *STPI* State-Trait Personality Inventory

^*^ *p* <.05, ^**^ *p* <.01, ^***^p <.001

#### Irrational performance beliefs

The original English language version of the irrational Performance Beliefs Inventory (iPBI) [54] consists of 28 items, with seven items assigned to each dimension of irrational beliefs as conceptualized within REBT: PIB (e.g., “I must not be dismissed by my peers.”), LFT (e.g., “I cannot bear not getting better at what I do”), AWF (e.g., “It’s terrible if the members of my team do not respect me”), and DEP (e.g., “I am a loser if I do not succeed in things that matter to me”). Participants rate each item on a five-point scale from 1 (*strongly disagree*) to 5 (*strongly agree*). The iPBI has demonstrated reliability across various samples across numerous studies, including within working populations [54]; athletes and exercisers [29, 31].

#### Shortened general attitude and belief scale (SGABS)

The original English version of the SGABS [34] captures irrational beliefs using 22 items. The SGABS consists of six irrational beliefs subscales, namely ‘Need for achievement’ (4 items; e.g., “It’s unbearable to fail at important things, and I can’t stand not succeeding at them.”), ‘Need for approval’ (3 items; e.g., “When people who I want to like me, disapprove of me or reject me, I can’t bear their disliking me.”), ‘Need for comfort’ (4 items; e.g., “It’s unbearable being uncomfortable, tense or nervous and I can’t stand it when I am.”), ‘Demand for fairness’ (4 items; e.g., “I think it is terribly bad when people treat me with disrespect.”), ‘Self-downing’ (4 items; e.g., “If important people dislike me, it is because I am an unlikable bad person.”), and ‘Other downing’ (3 items; e.g. “I believe that if a person treats me very unfairly, they are bad and worthless.”). All subscales can be summed into one ‘Total Irrationality’ scale. Participants indicated on a 5-point scale ranging from 1 (*strongly disagree*) to 5 (*strongly agree*), to the extent that they agreed with each of the 26 statements. Cronbach’s alpha in the original sample was α =.79 (Need for achievement’), α =.79 (Need for approval’), α =.80 (‘Need for comfort’), α =.80 (‘Demand for fairness’), α =.83 (‘Self-downing’), and α =.61 (‘Other downing’) [34].

#### State-trait personality inventory (STPI)

The original English version of the STPI [46] captures trait ‘Anxiety’ (e.g., “I feel nervous and restless.”), ‘Anger’ (e.g., “I am quick tempered.”), ‘Depression’ (e.g., “I feel gloomy.”), and ‘Curiosity’ (e.g., “I feel like exploring my environment.”) with 10 item each. However, for the current study we omitted ‘Curiosity’ due to a lack of justifiable association with irrational beliefs. Participants rated their experience on a 4-point scale ranging from 1 (*almost never*) to 4 (*almost always*). Cronbach’s α in the original sample was α ≥.81 (Depression), α ≥.89 (Anger) and, α ≥.86 (Anxiety) [46].

### Procedure

Participants for our study were recruited in two ways: through private contacts and paid advertising on Facebook for account holders residing in Malaysia and Singapore. Between June 2017 and the February 2018, participants completed a series of questionnaires online via Qualtrics (Qualtrics, Provo, UT) that included iPBI, SGABS, and STPI as well as demographic questions. Ethical approval was granted from the local Review Board of the second author’s institution. To be eligible, participants had to be at least 15 years old, currently reside in Malaysia or Singapore at the time of the survey and have spent the first 12 years of their life in Malaysia or Singapore. Participants were informed about the nature and the procedure of the study and gave consent before completing the questionnaires. Participation was voluntary and participants received no incentives.

### Data analyses

We performed statistical analyses using R Studio [42].

#### Data screening

First, the missing data was minimal because we used the “forced response” option in Qualtrics. Thus only less than 10% of the data in the dataset were missing (missing values: *n* = 1), mean imputation for the missing data point was applied [58]. Second, we checked our data for univariate and multivariate normal distribution using Shapiro–Wilk-test for univariate normality and Mardia’s coefficient for multivariate normality (“mvn”-package; [33]). We separated the analyses by language (*i.e.*, Bahasa Malaysia and Mandarin). The analyses revealed neither a normal distribution for the individual items nor a multivariate normal distribution of the data (*Bahasa Malaysian data*: Mardia Kurtosis: 23.38,* p* <.001; *Mandarin data:* Mardia Kurtosis: 24.69,* p* <.001; [36, 50]). Thus, we chose a robust maximum likelihood estimation (MLR) that computes standard errors and model fit indices that are robust in relation to the relative non-normality of observations for subsequent confirmatory factor analysis (CFA) [28]. Third, we screened data for outliers (standardized *z*-values > 3.29; [35, 50]), and winsorized outliers (*Bahasa Malaysian data*: *n* = 25 from 6,692 cases < 0.01%; *Mandarin data*: *n* = 21 from 5,040 cases < 0.01%).

#### Confirmatory factor analysis (CFA)

We conducted two separate CFAs with the 28 translated iPBI items to test the four-factor structure of the measure in our two subsamples and to evaluate factor loadings, error variances, and modification indices. By means of additional exploratory analyses, we examined whether the items included in the already existing Korean and Thai versions of the iPBI (iPBIK and T-iPBI) could be applied to our data and provide a better model fit (for more details see “Exploratory Analyses”). To test the different models, we proceeded as follows: First, we inspected fit indices for each model to evaluate the goodness of model fit [32]. In detail, we reported the χ^2^-test statistic, the Root Mean Square Error of Approximation (RMSEA) and its confidence interval (90% CI), as well as the Standardized Root Mean Square Residual (SRMR), the Comparative Fit Index (CFI), and the Tucker Lewis Index (TLI). The following evaluation criteria were applied: RMSEA-values less than.08 indicate an acceptable model and less than.06 indicate a good model [27]. For the SRMR index, values should be <.05 for a good fit and <.10 for an acceptable fit. Regarding CFI und TLI index, values >.90 are indicative of a good model fit [27]. To achieve a good fitting model, at least two fit indices should show an acceptable fit [27]. Second, we calculated the Akaike Information Criterion (AIC) to compare the tested models. Since the AIC does not have a specific range of acceptable values, we assumed that the lower the value, the more likely it is that the model can be replicated in other samples. Finally, we used the CFI difference (ΔCFI) and the scaled chi-square difference computation to statistically compare the models. A ΔCFI <.01 is considered as no difference (Kline 2005) and the following applies to the scaled chi-square difference computation: If the difference chi-square is statistically significant, then the null hypothesis is rejected and the baseline model (= less restrictive model with more estimated parameters and smaller *df*) fits the data better than the nested comparison model (= more restricted model with fewer estimated parameter and with larger *df*). If the chi-square difference is not statistically significant, the opposite is the case [43]. In addition, we only retained items that had factor loadings ≥.40 on the intended factor [24].

#### Correlational analyses

We conducted three independent Pearson’s correlation analyses to examine (a) the inter-correlations of the iPBI-Malay and iPBI-Mandarin subscales, (b) the construct validity by correlating their subscales with an existing measure of irrational beliefs (convergent/divergent validity), and (c) the concurrent validity with theoretically related constructs. For the last two analyses mentioned, we correlated subscales of the iPBI-Malay and iPBI-Mandarin with subscales of the SGABS [34] and STPI [46]. We corrected* p*-values for multiple comparisons using Holm’s method for each analysis. We applied the following criteria to evaluate the correlation coefficients: very high (≥.90), high (≥.70), moderate (≥.50), and weak (<.50) [26]. Regarding the correlations with SGABS, we expected that all subscales would be positively correlated with dimensions of the iPBI-Malay and iPBI-Mandarin. Furthermore, we assumed that ‘Anxiety’, ‘Anger’ and ‘Depression’ would be positively correlated with iPBI-Malay and iPBI-Mandarin dimensions.

#### Scale reliability

We computed Cronbach’s alpha coefficients (Cronbach’s α) as measures of internal consistency of the iPBI-Malay and iPBI-Mandarin and their dimensions. Coefficients greater than.70 indicate good test score reliability [40].

#### Potential population-based differences

To further investigate population-based differences, we correlated participants’ age with irrational performance beliefs scores and used independent* t*-tests to examine differences between women and men. Beforehand, we checked the requirements for the application via Shapiro–Wilk-Test for testing the assumption of normality (*p* >.05) and via Levene’s Test for checking the homogeneity of variance (*p* >.05). In case of a non-parametric distribution, we reported the significance of Wilcox signed-rank test as robust alternative for an independent *t*-test (*p*_wilcox_; [21]). According to previous findings (e.g., [38, 54]) we assumed that women would report a greater irrational belief than men and that younger participants would report greater irrational performance beliefs than older participants.

## Results

### Subsample 1 (iPBI-Mandarin)

#### Confirmatory factor analysis

**Fig. 1:**
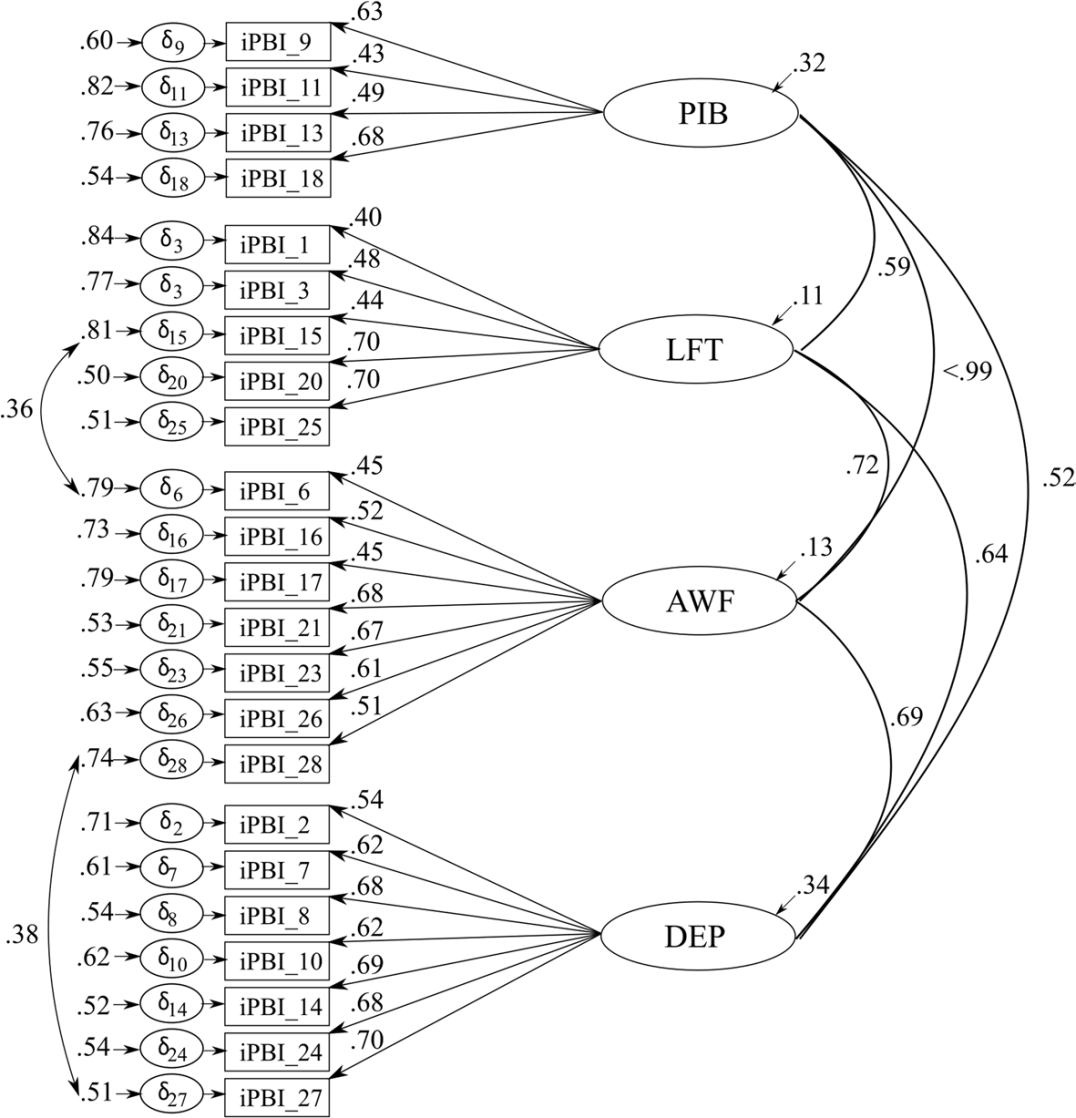
4-Factor 23-item solution of the iPBI-Mandarin with standardized factor loadings and error variances. Note. iPBI = Irrational Performance Beliefs Inventory, PIB = Primary irrational Beliefs, LFT = Low-frustration-tolerance, AWF = Awfulization, DEP = Depreciation, ձ = Standardized error variances

Results of the initial CFA revealed an unacceptable fit to the expected four-factor solution (Model 1), *N* = 180, *χ*^2^(344) = 573.521, *p* <.001, CFI =.815, TLI =.797, RMSEA =.064[.055,.073], SRMR =.075, AIC = 9750.343. Therefore, through a careful examination of the initial model, we conducted a further CFA, in which we excluded three PIB items (Item 4: “Decisions that affect me must be justified.“, Item 5: “I have to be viewed favorably by people that matter to me.”, and Item 22: “I need my manager/coach to act respectfully towards me.”) and two LFT items (Item 12: “I can’t bear not succeeding in things that are important to me.”, Item 19: “I can’t stand failing in things that are important to me.”) due to low factor loadings (.24,.27,.10,.21,.29)^1^. In addition, we carried out a third and a fourth CFA, in which we considered stepwise the proposed modification in the form of a covariation of the residuals of Item 6 and 15, as well as Item 27 and 28. The result of the final CFA (Model 2) showed improved model fit, closer to the recommended model fit indices, *N* = 180, χ^2^(222) = 376.788, *p* <.001, CFI =.866, TLI =.847, RMSEA =.066[.054,.077], SRMR =.074, AIC = 8606.402. However, it must be considered that the correlation between the PIB and the AWF dimension was >.99. Standardized factor loadings ranged from.402 to.704. Error variances were between.504 and.838 (see Fig. 1). Final items of the iPBI-Mandarin are presented in Table S2 in the supplement.

#### Exploratory analyses

As the model fit of the iPBI-Mandarin version was not fully satisfactory, we examined whether the items in the already existing Korean and Thai versions of the iPBI (iPBIK and T-iPBI) could be applied to our data and provide a better model fit. Therefore, we first used the 20-item Thai version, consisting of the 4 PIB items (PIB Item 2–5), 4 LFT items (LFT Item 2, 4, 6, 7), 6 AWF items (AWF Item 1–6), and 6 DEP items (DEP Item 2–7) of the original iPBI [54]. Second, we then used the 20-Item Korean version consisting of 2 PIB items (PIB Item 4 & 6), 5 LFT items (LFT Item 3–7), 7 AWF items (complete AWF dimension), and 6 DEP items (DEP Item 2–7) of the original iPBI [54]. Third, we compared the different models using ΔCFI and the scaled chi-square difference test to determine the best model fit for our sample. Results of the different CFAs are displayed in Table 2.

**Table 2:** Model fit indices of the different CFA models separated by subsample and applied iPBI version

	χ2	*df*	CFI	TLI	RMSEA [90% CI]	SRMR	AIC
*Mandarin speaking sample (N* = *180)*							
*23-item Mandarin version (iPBI-Mandarin)*							
Model 1	573.521, *p* <.001	344	.815	.797	.064[.055,.073]	.074	9750.343
Model 2	376.788, *p* <.001	222	.866	.847	.066[.054,.077]	.074	8606.402
*20-item Thai version*							
Model 3	274.001, *p* <.001	164	.877	.857	.065[.051,.078]	.072	7349.015
Model 4	255.303, *p* <.001	163	.897	.880	.060[.045,.073]	.070	7329.257
*20-items Korean version*							
Model 5	295.503, *p* <.001	164	.853	.830	.072[.058,.085]	.076	6957.458
Model 6	255.226, *p* <.001	162	.896	.878	.061[.046,.075]	.072	6914.350
*Malay speaking sample (N* = *239)*							
*25-item Malay version (iPBI-Malay)*							
Model 1	819.714, *p* <.001	344	.763	.740	.079[.072,.086]	.081	16,369.541
Model 2	562.134, *p* <.001	266	.841	.821	.072[.064,.080]	.077	14,383.913
*20-item Thai version*							
Model 3	431.716, *p* <.001	164	.812	.782	.088[.078,.098]	.076	12,023.156
Model 4	336.468, *p* <.001	161	.877	.855	.072[.061,.082]	.075	11,921.661
*20-items Korean version*							
Model 5	486.457, *p* <.001	164	.781	.747	.099[.089,.109]	.076	11,611.028
Model 6	372.280, *p* <.001	160	.857	.830	.081[.070,.091]	.067	11,480.923

*χ*^*2*^ chi-square test statistic, *RMSEA* Root Mean Square Error of Approximation, *CI* Confidence Interval, *CFI* Comparative Fit Index, *TLI* Tucker-Lewis Index, *SRMR* Standardized Root Mean Square Residual, *AIC* Akaike Information Criterion

The solution of the initial 20-item Thai version (Model 3) was not sufficient, χ^2^(164) = 274.001, *p* <.001, CFI =.877, TLI =.857. After we have allowed the error variances of Item 15 and 6 to co-vary, the model (Model 4) showed an acceptable fit, χ^2^(163) = 255.303, *p* <.001, CFI =.897, TLI =.880. Standardized factor loadings ranged from.431 to.736 with one exception (Item 5 =.295). Error variances were between.459 and.913. Comparing the 23-item iPBI-Mandarin version (Model 2) and Model 4, the scaled chi-square difference test (Δχ^2^ = 83.368, *df* = 38, *p* <.001) and CFI differences (ΔCFI =.031) indicated that Model 4 offers a superior model fit. In addition, the initial 20-item Korean version (Model 5) was not an acceptable fit, χ^2^(164) = 295.503, *p* <.001, CFI =.853, TLI =.830. After the covariation of the error variances of Item 6 and 15 and Item 27 and 28, the model (Model 6) was an acceptable fit, χ^2^(162) = 255.226, *p* <.001, CFI =.896, TLI =.878. However, it must be considered that the correlation between the PIB and the AWF dimension was >.99. Standardized factor loadings ranged from.407 to.727 but two items did not reach the required factor loading of.40 (Item 12 =.171 and Item 19 =.306). Error variances were between.472 and.971. Comparing the 23-item iPBI-Mandarin version (Model 2) and Model 6, the scaled chi-square difference test (Δχ^2^ = 85.359, *df* = 39, *p* <.001) and CFI differences (ΔCFI =.030) indicated that Model 6 offers a superior model fit. Finally, we compared Model 4 and Model 6 with each other. A CFI difference of.001 indicated that both models have a similarly sufficient model fit (the scaled chi-square difference test was not applicable).

#### Correlational analyses

First, we calculated inter-correlations between the subscales of the respective iPBIs. Results are summarized in Table 1. Second, we tested construct validity through correlations between the three different iPBI versions (i.e., 23-item iPBI-Mandarin, applied 20-item Thai version, and applied 20-item Korean version) and SGABS dimensions. Results indicated significant low to medium positive correlations between the dimensions of the iPBIs (i.e., iPBI-Mandarin: *r* =.23–.68; Thai version: *r* =.25–.67; Korean version: *r* =.20–.67) and those of the SGABS measuring irrationality. One exception was the correlations between the PIB of the 20-item Korean version and one subscale of the SGABS, which was not significant. Furthermore, the largest correlation was between the ‘Total irrationality’ scale and the respective iPBI composite scores (i.e., iPBI-Mandarin: *r* =.73; Thai version: *r* =.72; Korean version: *r* =.72). Third, we tested concurrent validity, of the iPBIs through correlations with trait subscales of the STPI. The results indicated low correlations between the iPBI-Mandarin dimensions and the STPI, with only 3 out of 4 correlations with the ‘Anger’ and ‘Anxiety’ subscales being significant (see Table 1). No significant correlations were observed for the ‘Depression’ subscale. Therefore, our hypothesis can only be partially confirmed. Participants who reported more irrational beliefs had higher trait anxiety and anger scores but not higher depression scores.

Regarding the correlations with the dimension of the Thai version, there were weak but significant correlations with the ‘Anxiety’ and ‘Anger’ subscales, except for the AWF dimension. Again, there were no significant correlations with the ‘Depression’ subscale. The same pattern of results applied to the correlations with the Korean version.

#### Scale reliability

Cronbach’s α coefficients were between.62 and.84 (see first third of Table 1) for iPBI-Mandarin dimensions and.89 for the composite score of the 23-item iPBI-Mandarin. With α <.70, two dimensions (i.e., PIB and AWF) were not ‘good’, but still in an acceptable range [40]. The same pattern applies to the Cronbach’s α coefficients of the dimensions of the 20-item Thai version and 20-item Korean version. Cronbach’s α coefficients were between.51 and.83 (20-item Thai version) and between.57 and.83 (20-item Korean version) with both the PIB and the LFT dimension with an alpha <.70. The α of the composite scores of both versions were.88.

#### Population-based differences

Table 3 (upper part) presents descriptive statistics and results of the test statistic in terms of differences between participants’ sex in the respective iPBI dimension separated by our three different versions (i.e., 25-item iPBI-Mandarin, applied 20-item Thai version, and applied 20-item Korean version), as well as the results of the correlational analyses between iPBI dimensions and participants’ age. Results of the independent *t*-test showed only a trend towards a significant difference between woman and men in AWF (both *p*_wilcox_ =.06) for the 23-item iPBI-Mandarin and the 20-item Korean version. Furthermore, correlational analyses between participants’ age and all iPBI dimensions were non-significant. Thus, our hypotheses regarding sex and age could not be confirmed in Subsample 1, regardless of the iPBI version selected.

**Table 3:** Descriptive statistics and effect size differences of iPBI subscales separated by different criteria

		**PIB**	**LFT**	**AWF**	**DEP**	**iPBI Composite score**
*iPBI-Mandarin*						
Age	*r*	.11	-.12	-.01	-.04	-.03
Males (*n* = 118)	*M* (SD)	3.50 (0.46)	3.50 (0.48)	3.35 (0.49)	2.80 (0.70)	3.22 (0.45)
Females (*n* = 62)	*M* (SD)	3.52 (0.45)	3.52 (0.50)	3.49 (0.45)	2.84 (0.73)	3.29 (0.46)
*t*-test statistic		*p*_wilcox_ =.76	*p*_wilcox_ =.77	*p*_wilcox_ =.06	*p*_wilcox_ =.43	*p*_wilcox_ =.21,
Effect size		*r*_*w*_ =.02	*r*_*w*_ =.02	*r*_*w*_ =.14	*r*_*w*_ =.06	*r*_*w*_ =.09
*Thai version*						
Age	*r*	.12	-.11	-.03	-.03	-.03
Males (*n* = 118)	*M* (SD)	3.52 (0.42)	3.47 (0.50)	3.42 (0.50)	2.80 (0.71)	3.26 (0.43)
Females (*n* = 62)	*M* (SD)	3.52 (0.41)	3.53 (0.52)	3.52 (0.45)	2.87 (0.74)	3.32 (0.45)
*t*-test statistic		*p*_wilcox_ =.98	*p*_wilcox_ =.38	*p*_wilcox_ =.17	*p*_wilcox_ =.42	*p*_wilcox_ =.27
Effect size		*r*_*w*_ <.01	*r*_*w*_ =.06	*r*_*w*_ =.10	*r*_*w*_ =.06	*r*_*w*_ =.08
*Korean version*						
Age	*r*	.03	-.08	-.01	-.02	-.03
Males (*n* = 118)	*M* (SD)	3.64 (0.43)	3.60 (0.37)	3.35 (0.49)	2.80 (0.71)	3.28 (0.43)
Females (*n* = 62)	*M* (SD)	3.63 (0.42)	3.61 (0.39)	3.49 (0.45)	2.87 (0.74)	3.35 (0.44)
*t*-test statistic		*p*_wilcox_ =.78	*p*_wilcox_ =.63	*p*_wilcox_ =.06	*p*_wilcox_ =.42	*p*_wilcox_ =.18
Effect size		*r*_*w*_ =.02	*r*_*w*_ =.03	*r*_*w*_ =.14	*r*_*w*_ =.06	*r*_*w*_ =.10

*p*-values were corrected for multiple comparisons using Holm’s method, *r* = Pearson correlation coefficient; effect size: *r*_*w*_ = effect size for robust *t*-test according to Wilcox, *d* = Cohen’s *d*

*PIB* Primary irrational Beliefs, *LFT* Low-frustration-tolerance, *AWF* Awfulization, *DEP* Depreciation

^*^ *p* <.05, ^**^ *p* <.01, ^***^p <.001

### Results Subsample 2 (iPBI-Malay)

#### Confirmatory factor analysis iPBI

**Fig. 2:**
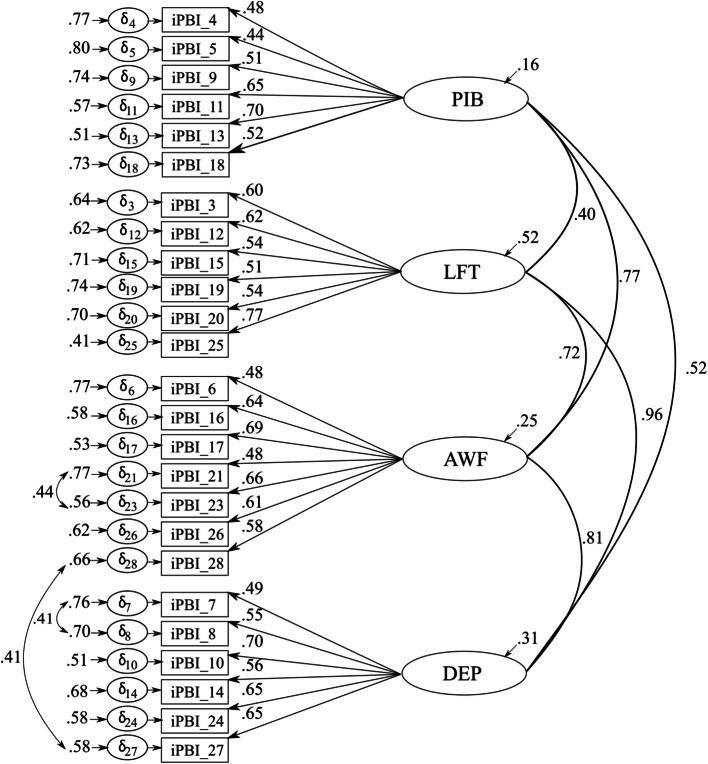
4-Factor 25-item solution of the iPBI-Malay with standardized factor loadings and error variances. Note. iPBI = Irrational Performance Beliefs Inventory, PIB = Primary irrational Beliefs, LFT = Low-frustration-tolerance, AWF = Awfulization, DEP = Depreciation, ձ = Standardized error variances

To test the four-factor structure of the iPBI-Malay in Subsample 2 using CFAs, we proceeded in a similar way to Subsample 1. Again, results of the initial CFA revealed an unacceptable fit to the expected four-factor solution (Model 1), *N* = 239, *χ*^2^(344) = 819.714, *p* <.001, CFI =.763, TLI =.740, RMSEA =.079[.072,.086], SRMR =.081, AIC = 16,369.541. One PIB item (Item 22: “I need my manager/coach to act respectfully towards me”), one LFT item (Item 1: “I can’t bear not being given chances.”), and one DEP item (Item 2: “If decisions that affect me are not justified, it shows that I am worthless.”) showed low factor loadings (.23,.11, and.31). We have therefore excluded these for further analyses and calculated a second CFA^2^. In addition, we carried out further CFAs, in which we considered stepwise the proposed modification in the form of a covariation of the residuals of Item 21 and 23, Item 7 and Item 8, as well as Item 28 and Item 27. The result of the final CFA (Model 2) showed a better model fit compared to the initial model and is closer to the recommended model fit indices. *N* = 239, *χ*^2^(266) = 562.134, *p* <.001, CFI =.841, TLI =.821, RMSEA =.072[.064,.080], SRMR =.077, AIC = 14,383.913. Standardized factor loadings ranged from.443 to.771. Error variances were between.406 and.771 (see Fig. 2). Final items of the iPBI-Malay are presented in Table S3 in the supplement.

#### Exploratory analyses

For the same reasons as with Subsample 1, we also carried out further exploratory analyses with our data and the existing versions of the iPBI from the Asian region (i.e., T-iPBI and iPBIK). We followed the same procedure as described under ‘Exploratory analyses’ in the ‘Results’ section of Subsample 1.

Regarding the results of the CFA with the initial 20-item Thai version (Model 3), the model fit was not sufficient, *χ*^2^(164) = 431.716, *p* <.001, CFI =.812, TLI =.782. After we have allowed the error variances of Item 21 and 23, Item 7 and 8 s well as Item 23 and 27 to co-vary, the model (Model 4) showed improved model fit, closer to the recommended model fit indices, *χ*^2^(161) = 336.468, *p* <.001, CFI =.877, TLI =.855. Standardized factor loadings ranged from.425 to.822. Error variances were between.325 and.820. Comparing the Model 2 and Model 4, the scaled chi-square difference test (Δ*χ*^2^ = 225.720, *df* = 105, *p* <.001) and CFI differences (ΔCFI =.036) indicated that Model 4 offers a better model fit.

In addition, the initial 20-item Korean version (Model 5) was not an acceptable fit, χ^2^(164) = 486.457, *p* <.001, CFI =.781, TLI =.747. After the covariation of the error variances of the following item combinations: Item 21 and 23, Item 7 and 8, Item 16 and 17, and Item 27 and 28, the model fit increased (Model 6) closer to an acceptable fit, *χ*^2^(160) = 372.280, *p* <.001, CFI =.857, TLI =.830. Standardized factor loadings ranged from.489 to.750. Error variances were between.438 and.761. Comparing the Model 2 and Model 6, only the scaled chi-square difference test (Δ*χ*^2^ = 185.47, *df* = 106, *p* <.001) indicated that Model 6 offers a better model fit (ΔCFI =.016). Finally, we compared Model 4 and Model 6 with one another. A CFI difference of.020 indicated that Model 4 offers a better model fit (the scaled chi-square difference test was not applicable).

#### Correlational analyses

Results of the (inter-) correlations are summarized in Table 4. The correlations between the iPBI dimensions of the different iPBI versions (i.e., iPBI-Malay, applied Thai and Korean version and the SGABS subscales for the irrationality-related subscales were significant in a low to medium range, except for ‘Need for Achievement’ and PIB and ‘Self-Downing’ and PIB. In addition, the highest correlation was between the ‘Total irrationality’ subscale and the iPBI composite score for the iPBI-Malay as well as the Thai version (both *r* =.70) and the Korean version (*r* =.69). Thus, except for the correlations between PIB and the two subscales ‘Need for Achievement’ and ‘Self-Downing’, all correlations were in line with our expectations regarding the irrationality-related subscales of the SGABS. Next, correlational analyses between dimensions of the different iPBI versions and STPI subscales showed weak positive, but significant associations for ‘Anxiety’, ‘Anger’ and ‘Depression’ (except for the associations between ‘Anxiety’ and PIB, and ‘Depression’ and PIB). In addition, only a negative correlation between PIB and ‘Depression’ in the applied Korean version was striking because of its highly unexpected and counter-theoretical negative correlation. Therefore, our hypothesis can be largely confirmed in Subsample 2, in the sense that participants who report more irrational beliefs have higher ‘Anxiety’, ‘Anger’ and ‘Depression’ scores.

**Table 4:** Descriptive statistics, inter-correlations for the iPBI dimensions, and correlations for the Bahasa Malaysia speaking sample

	***M* (*SD*)**	**α**	**PIB**	**LFT**	**AWF**	**DEP**	**iPBI Composite score**
iPBI-Malay							
Primary irrational beliefs (PIB)	4.05 (0.53)	.69	-				
Low frustration tolerance (LFT)	3.88 (0.64)	.76	.29^***^	-			
Awfulization (AWF)	3.85 (0.63)	.80	.52^***^	.60^***^	-		
Depreciation	3.41 (0.79)	.80	.33^***^	.69^***^	.65^***^	-	
iPBI Composite score	3.80 (0.52)	.90	-	-	-	-	-
* SGABS*							
Need for achievement	3.58 (0.62)	.51	.16	.59^***^	.42^***^	.53^***^	.55^***^
Need for approval	3.40 (0.72)	.55	.42^***^	.46^***^	.56^***^	.50^***^	.61^***^
Need for comfort	2.91 (0.60)	.49	.33^***^	.27^***^	.26^***^	.29^***^	.35^***^
Demand on fairness	3.69 (0.63)	.68	.49^***^	.46^***^	.65^***^	.48^***^	.65^***^
Self-downing	2.62 (0.72)	.61	.13	.27^***^	.25^***^	.38^***^	.33^***^
Other downing	3.20 (0.80)	.54	.31^***^	.32^***^	.37^***^	.29^***^	.40^***^
Total irrationality	3.23 (0.46)	.83	.44^***^	.58^***^	.61^***^	.60^***^	.70^***^
* STPI*							
Anxiety	23.33 (4.58)	.77	.14	.31^***^	.31^***^	.45^***^	.39^***^
Anger	21.81 (5.10)	.82	.30^***^	.27^***^	.32^***^	.36^***^	.39^***^
Depression	22.01 (4.58)	.79	.04	.22^**^	.18^*^	.37^***^	.27^***^
Thai version							
Primary irrational beliefs (PIB)	4.11 (0.59)	.67	-				
Low frustration tolerance (LFT)	3.68 (0.77)	.71	.22^***^	-			
Awfulization (AWF)	3.84 (0.63)	.76	.48^***^	.53^***^	-		
Depreciation	3.41 (0.79)	.80	.30^***^	.66^***^	.62^***^	-	
iPBI Composite score	3.73 (0.56)	.88	-	-	-	-	-
* SGABS*							
Need for achievement	3.58 (0.62)	.51	.12	.55^***^	.39^***^	.53^***^	.53^***^
Need for approval	3.40 (0.72)	.55	.38^***^	.45^***^	.55^***^	.50^***^	.60^***^
Need for comfort	2.91 (0.60)	.49	.31^***^	.29^***^	.27^***^	.29^***^	.36^***^
Demand on fairness	3.69 (0.63)	.68	.43^***^	.44^***^	.64^***^	.48^***^	.63^***^
Self-downing	2.62 (0.72)	.61	.13	.30^***^	.24^**^	.38^***^	.35^***^
Other downing	3.20 (0.80)	.54	.27^***^	.31^***^	.35^***^	.29^***^	.38^***^
Total irrationality	3.23 (0.46)	.83	.39^***^	.57^***^	.59^***^	.60^***^	.70^***^
* STPI*							
Anxiety	23.33 (4.58)	.77	.15	.31^***^	.30^***^	.45^***^	.41^***^
Anger	21.81 (5.10)	.82	.29^***^	.25^***^	.32^***^	.36^***^	.39^***^
Depression	22.01 (4.58)	.79	.04	.25^***^	.17^*^	.37^***^	.29^***^
Korean version							
Primary irrational beliefs (PIB)	4.01 (0.64)	-	-				
Low frustration tolerance (LFT)	3.99 (0.61)	.72	.36^***^	-			
Awfulization (AWF)	3.85 (0.63)	.80	.52^***^	.62^***^	-		
Depreciation (DEP)	3.41 (0.79)	.80	.32^***^	.67^***^	.65^***^	-	
iPBIK Composite score	3.77 (0.57)	.90	-	-	-	-	-
* SGABS*							
Need for achievement	3.58 (0.62)	.50	.16	.59^***^	.42^***^	.53^***^	.56^***^
Need for approval	3.40 (0.72)	.55	.43^***^	.45^***^	.56^***^	.50^***^	.60^***^
Need for comfort	2.91 (0.60)	.49	.26^***^	.24^**^	.26^***^	.29^***^	.32^***^
Demand on fairness	3.69 (0.63)	.69	.46^***^	.46^***^	.66^***^	.48^***^	.63^***^
Self-downing	2.62 (0.72)	.61	.12	.24^**^	.25^***^	.38^***^	.33^***^
Other downing	3.20 (0.80)	.54	.28^***^	.31^***^	.37^***^	.29^***^	.38^***^
Total irrationality	3.23 (0.46)	.82	.41^***^	.56^***^	.61^***^	.61^***^	.69^***^
* STPI*							
Anxiety	23.33 (4.58)	.77	.06	.27^***^	.31^***^	.44^***^	.39^***^
Anger	21.81 (5.10)	.82	.21^**^	.23^**^	.32^***^	.35^***^	.36^***^
Depression	22.01 (4.58)	.79	-.01	.19^*^	.18^*^	.37^***^	.27^***^

*p*-values were corrected for multiple comparisons using Holm’s method

*M* Mean, *SD* Standard deviation, *α* Cronbach’s alpha, *iPBI* irrational Performance Beliefs Inventory, *SGABS* Shortened General Attitude and Belief Scale, *STPI* State-Trait Personality Inventory

^*^ *p* <.05, ^**^ *p* <.01, ^***^p <.001

#### Scale reliability

Cronbach’s α coefficients were all between.70 and.80 (see upper part Table 1) for iPBI-dimensions, and thus within the recommended range. Two exceptions are Cronbach’s α for the PIB dimension of the iPBI-Malay and Thai version, which is still in the good range ≥.67. Moreover, Cronbach’s alpha for the composite scores were.90 for the 25-item iPBI-Malay and the Korean version and.88 for Thai version.

#### Population-based differences

Table 5 presents descriptive statistics and results of the test statistic in terms of differences between participants’ sex and the respective iPBIs dimension as well as the results of the correlational analysis between iPBIs dimensions and participants’ age. In contrast to Subsample 1, the results of the independent *t*-test showed significant differences between women and men in AWF, DEP and the iPBI composite scores, with women having higher scores than men on the dimensions mentioned. Furthermore, correlational analyses between participants’ age and dimensions of all iPBI versions were significant (except for PIB) and showed in the expected direction. Thus, our hypotheses regarding gender and age were largely confirmed in Subsample 2.

**Table 5:** Descriptive statistics and effect size differences of iPBI subscales separated by different criteria

		**PIB**	**LFT**	**AWF**	**DEP**	**iPBI Composite score**
*iPBI-Malay*						
Age	*r*	-.01	-.25^***^	-.22^**^	-.33^***^	-.27^***^
Males (*n* = 162)	*M* (SD)	4.01 (0.57)	3.86 (0.66)	3.76 (0.65)	3.32 (0.80)	3.73 (0.53)
Females (*n* = 77)	*M* (SD)	4.13 (0.45)	3.91 (0.58)	4.03 (0.53)	3.61 (0.74)	3.92 (0.48)
*t*-test statistic		*p*_wilcox_ =.15	*p*_wilcox_ =.37	*p*_wilcox_ <.01	*p*_wilcox_ <.01	*t*(163.84) = −2.71, *p* <.01
Effect size		*r*_*w*_ =.09	*r*_*w*_ =.06	*r*_*w*_ =.20	*r*_*w*_ =.18	*d* = 0.36
*Thai version*						
Age	*r*	-.04	-.28^***^	-.20^**^	-.33^***^	-.29^***^
Males (*n* = 162)	*M* (SD)	4.06 (0.62)	3.66 (0.79)	3.76 (0.66)	3.32 (0.80)	3.67 (0.56)
Females (*n* = 77)	*M* (SD)	4.19 (0.51)	3.74 (0.70)	4.01 (0.53)	3.61 (0.74)	3.87 (0.52)
*t*-test statistic		*p*_wilcox_ =.14	*p*_wilcox_ =.39	*p*_wilcox_ <.01	*p*_wilcox_ <.01	*t*(161.87) = −2.75, *p* <.01
Effect size		*r*_*w*_ =.09	*r*_*w*_ =.05	*r*_*w*_ =.18	*r*_*w*_ =.18	*d* = 0.37
*Korean version*						
Age	*r*	<.01	-.22^**^	-.22^**^	-.33^***^	-.28^***^
Males (*n* = 162)	*M* (SD)	3.97 (0.67)	3.97 (0.63)	3.76 (0.65)	3.32 (0.80)	3.70 (0.58)
Females (*n* = 77)	*M* (SD)	4.10 (0.55)	4.03 (0.56)	4.03 (0.53)	3.61 (0.74)	3.91 (0.50)
*t*-test statistic		*p*_wilcox_ =.27	*p*_wilcox_ =.35	*p*_wilcox_ <.01	*p*_wilcox_ <.01	*t*(171.37) = −2.82, *p* <.01
Effect size		*r*_*w*_ =.07	*r*_*w*_ =.06	*r*_*w*_ =.20	*r*_*w*_ =.18	*d* = 0.39

*p*-values were corrected for multiple comparisons using Holm’s method, *r* = Pearson correlation coefficient; effect size: *r*_*w*_ = effect size for robust *t*-test according to Wilcox, *d* = Cohen’s *d*

^*^ *p* <.05, ^**^ *p* <.01, ^***^p <.001

## General discussion

In this study we aimed to translate, and validity test the irrational Performance Beliefs Inventory in Mandarin and Bahasa Malaysia languages, yielding the iPBI-Mandarin and iPBI-Malay. Initially, we carried out a detailed translation process to develop these two new versions of the iPBI, before testing the four-factor structure of the new measures in both Mandarin and Bahasa Malaysia language speaking subsamples through CFA. In addition, we took further steps to assess the reliability and validity of the iPBI-Malay and iPBI-Mandarin, by (a) investigating their convergent and divergent validity using the different subscales of the SGABS, (b) examining their concurrent validity with correlations with participants’ anxiety, anger, and depression scores, (c) determining internal consistencies (Cronbach’s α) of their subscales and composite scores, and (d) testing the validity of the iPBI through sex comparisons and correlations with participants’ age. To try to arrive at the best model fit for the new Bahasa Malaysia and Mandarin items, we also exploratively applied the existing Thai and Korean versions of the iPBI (T-iPBI and iPBIK) to our sample and compared them with the iPBI-Mandarin and iPBI-Malay versions. In other words, we used the item composition and factor structure of the T-iPBI and iPBIK to test the extent to which the new Bahasa Malaysia and Mandarin items fit the Thai and Korean versions.

After multiple CFAs, we arrived at a 23-item Mandarin and 25-item new Bahasa Malaysia version, both of which showed an unsatisfactory model fit, which could be attributed to cultural differences between Western and Eastern cultures (for a detailed explanation see end of discussion part). This is also evident, for example, in the removed items. Statements like “Decisions that affect me must be justified”, or “I can’t bear not being given chances” contrast with the indirect communication styles of the community, where meanings are conveyed subtly and “face” is carefully preserved to avoid embarrassment or shame [22, 59]. In addition, those statements can be seen as confrontational and as a direct challenge to the judgment of elders or people of higher rank, which is considered disrespectful in collectivist societies [30]. Furthermore, this is supported by the fact that the overlap of the removed items between our versions and the other Eastern translations of the iPBI is very high, especially with regard to the PIB and LFT items [8, 9, 39]. Moreover, differences in the item solution between the Bahasa Malaysian and Mandarin versions, could be attributed to different linguistic landscapes that have arisen due to the formal status of Malay as the national language and the complexity of the Chinese language (including dialects and an increasing shift towards Mandarin) [59]. For instance, the Malay language (and culture) are centered on a unique national identity rooted in Malay customs, whereas the Chinese language (and culture) in Malaysia are an influential diaspora experience shaped by immigration and a strong connection to the traditions of Chinese ancestors, which are adapted within a multicultural Malaysian context [47]. This could be reflected in the meaning of the items, or more precisely, in the different interpretations of the items in both cultures, which could have led to the different item solutions. Finally, it has been shown that the 28-item version of the iPBI has not been fully replicated in any of the translations mentioned. Overall, our results and those of previous validation studies suggest that not all items work equally well in different cultures. It is therefore not surprising that both the Bahasa Malaysian and Mandarin items showed stronger model fit in our sample for the Thai and Korean versions. Nevertheless, model fit indices are still not fully within the recommended range (Thai version: Mandarin speaking sample: CFI =.897, TLI =.880; Bahasa Malaysia speaking sample: CFI =.877, TLI =.855) but comparable to the indices of other iPBI versions (cf., [8, 9, 54]. In addition, it should be kept in mind that these “golden rules” are subject to criticism, as they are very restrictive and hardly achievable when using personality questionnaires [37].

However, the factorial validity, in terms of correlations with convergent and divergent constructs (i.e., subscales of the SGABS), was largely confirmed and expected relationships were found between all versions of the iPBI and almost all subscales of the SGABS. But the picture was less clear for the tests of concurrent validity, whereby a mixed pattern of results emerged. For the iPBI-Mandarin our assumptions were only confirmed for ‘Anger’ and ‘Anxiety’ regardless of the iPBI model used, but for the iPBI-Malay significant positive correlations were found between four dimensions of the different iPBI versions and for ‘Anger’, ‘Anxiety’ and ‘Depression’. Thus, the results indicate that the iPBI-Malay and iPBI-Mandarin for the most part show good concurrent validity and are associated with symptoms of anxiety, anger, and depression consistent with previous iPBI validation studies [9, 38, 54] and a variety of non-performance specific irrational beliefs measures (e.g., [51]).

Concerning results that did not align with our expectations, it is striking that the correlations between PIB and ‘Anxiety’ and PIB and ‘Depression’ were not significant, neither for the iPBI-Malay nor the iPBI-Mandarin. In addition, the PIB dimension shows the weakest (but still sufficient) internal reliability in all iPBI versions, leading to the assumption that primary irrational beliefs might be interpreted and reported upon differently in our sample compared to Western samples. This suspicion is also perhaps reinforced by the non-optimal model fits of the various iPBI versions. Thus, it is perhaps more appropriate to generate new items from a more culturally attuned process of measurement development for specific Asian samples. Greater attention should be paid to cultural adaptations during the item development process to better tailor the measure to the underlying psyche and sociocultural aspects of the respective population. Therefore, validation of irrational performance beliefs measures in Asian populations should start from scratch to develop a more adequate measure adapted to the culture and to integrate REBT more purposefully into Eastern cultures (cf. [9]).

Concerning the population-related differences, the results for the Mandarin speaking sample showed neither a significant correlation between the iPBI dimensions and the age of the participants nor significant differences between men and women. This applies to all three iPBI versions and contradicts our hypothesis. In contrast, in the Bahasa Malaysia speaking sample, there were significant correlations between the iPBI dimensions and participants’ age in all three iPBI versions (i.e., iPBI-Malay, applied Thai and Korean version), indicating a decrease in irrational performance beliefs with age. This is in line with previous studies, which found that irrational performance beliefs were higher in younger participants [38, 53, 54]. Furthermore, results indicated significant differences between participants’ sex, with women scoring higher than men on the AWF and DEP dimension as well as on the composite scores, which is consistent with results from previous studies [38, 53, 54] and generally corresponds to findings from REBT research [7]. Accordingly, our hypotheses and the corresponding validity can be largely confirmed for the Bahasa Malaysia speaking sample, while this is less true for the Mandarin speaking sample. The divergence in findings between Mandarin speaking and Bahasa Malaysia speaking samples illustrates how we cannot assume that irrational beliefs develop and function in the same way cross-culturally. Also, irrational beliefs may reduce with age because as one is exposed to the litany of adversities that decorates the course of life, one learns that disadvantageous circumstances are not ‘the end of the world’ and that one can tolerate a great deal of ills without capitulating. But we cannot assume that the accumulation of life adversity shapes beliefs in the same way across different cultures and races, and more data is required in order to explore this.

### Limitations

The current paper has some shortcomings that need to be addressed. Firstly, we did not consider the social desirability of the participants’ responses when collecting data. In view of the cultural differences mentioned above, investigating this association could have helped to better interpret the results of the CFAs and embed them in the overall context of the study. Secondly, our study was limited by unequal distribution regarding the religion, the ethical affiliation, the educational level, and the gender of the participants (almost two thirds male participants). Consequently, future research should consider an equal distribution in terms of these characteristics that analyses of potential differences in a specific population can be carried out more adequately. In addition, our sample showed a very broad age distribution (16–50 years), encompassing participants at various stages of development. However, performance can be relevant in different contexts regardless of age. Furthermore, the original iPBI was developed to measure performance-related irrational beliefs in various contexts such as business, sports, science, medicine, and the military, and has been originally validated and applied in comparable samples to date (see [8, 9, 54]. Thirdly, we correlated residuals across factors in our various CFA models. Correlated residuals were specified between a small number of item pairs where modification indices indicated substantial localized misfit (MIS > 20). These items reflected domains of theoretical interdependence, suggesting that their shared variance could not be fully accounted for by the latent factors alone. This is further supported by high to very high covariations between the different iPBI dimensions (see Figs. 1 and 2). Modeling correlated uniqueness in such cases is consistent with best practice in CFA, as it reduces the risk of biased parameter estimates and inflated inter-factor correlations, while preserving construct validity and improving model fit. However, further studies are needed to verify (1) whether the separability of the dimensions from each other can still be strictly maintained or whether common variables between these factors need to be discussed, (2) or whether these are systematic methodological effects, issues caused by meaning or order of the items [3, 44]. Finally, for the criterion validation of our iPBI versions, we used the same measures as Turner et al. [54], namely the SGABS and the STPI. However, these measures have not yet been fully validated for the Malaysian population and were translated by us for the purpose of the study. Therefore, the results should be interpreted with caution and future studies should investigate the validity of the SGABS and STPI in the Malaysian population to obtain reliable results that will increase the applicability of the measures in Eastern populations and allow cross-cultural comparisons with Western countries.

## Conclusion

In summary, through exploration by factor analyses, the current paper arrives at two applicable, albeit imperfect, iPBIs for use in Bahasa Malaysia and Mandarin speaking populations. By applying a variety of four-factor models, it currently seems most promising to use the 20-item Thai version in Mandarin (due to fewer factor loading problems in the estimated model) and Bahasa Malaysia speaking samples (due to better model fit). Nevertheless, this study has shown that the application of Western measures is not always directly transferable to Eastern populations. To assist researchers and practitioners in assessing irrational beliefs in Malaysian and Mandarin populations, a measure should be developed from scratch that is closely adapted to the culture, mindset and characteristics of these populations. Only by doing this can we take into account cultural aspects and community differences in order to adequately capture irrational performance beliefs across cultures and establish REBT interventions as part of mainstream psychotherapy in Eastern populations. We therefore strongly recommend further work on the psychometric properties of the iPBI and research on irrational beliefs and their relationship to outcomes such as mental health and well-being. This is important to help identify cognitive risk factors for anxiety and depression in Malaysian and Mandarin speaking populations.

## Supplementary Information

Supplementary Material 1.

Supplementary Material 2

Supplementary Material 3

## Data Availability

The datasets generated and/or analyzed during the current study are available on OSF platform: https://osf.io/j2thd.

## Abbreviations

α: Cronbach's alpha
AIC: Akaike Information Criterion
AWF: Awfulizing
CFA: Confirmatory factor analysis
CFI: Comparative Fit Index
CI: Confidence Interval
*d*: Effect size for t-test according to Cohen
DEP: Depreciation
*df*: Degrees of freedom
iPBI: Irrational Performance Beliefs Inventory
iPBIK: Korean version of the irrational Performance Beliefs Inventory
LFT: Low-frustration tolerance
*M*: Mean
*N/*n: Sample size
PIB: Primary Irrational Belief
*r*: Pearson correlation coefficient
*r*_*w*_: Effect size for robust *t*-test according to Wilcox
REBT: Rational Emotive Behavior Therapy
RMSEA: Root Mean Square Error of Approximation
SD: Standard deviation
SGABS: Shortened General Attitude and Belief Scale
SRMR: Standardized Root Mean Square Residual
STPI: State-Trait Personality Inventory
T-iPBI: Thai version of the irrational Performance Beliefs Inventory
TLI: Tucker Lewis Index
TRAPD: Translation, Review, Adjudication, Pre-Testing and Documentation
χ2: Chi-square test statistic

## Footnotes

1 Model-fit after exclusion of six items: *N* = 180, *χ*^*2*^(224) = 415.696, *p* <.001, CFI =.833, TLI =.812, RMSEA =.073[.062,.084], SRMR =.077, AIC = 8647.388.

2 Model fit after exclusion of the three items: *N* = 239, χ2(269) = 662.958, *p* <.001, CFI =.787, TLI =.763, RMSEA =.083[.075,.091], SRMR =.080, AIC = 14495.600
