# Supplementary material for: Irrational beliefs in Bahasa Malaysia and Mandarin speaking populations: the cross-cultural validation of the irrational performance beliefs inventory
Source: BMC Psychol. 2025 Dec 1;13:1349. doi: 10.1186/s40359-025-03579-y (PMC12690909; doi:10.1186/s40359-025-03579-y)
Supplement: Supplementary file 1 — Supplementary Material 1. [file 40359_2025_3579_MOESM1_ESM.docx]

**Supplement**

Table S1

*Sample characteristics separated by subsamples and the total sample.*

|  | **Malay (n = 239)** | **Mandarin (n = 180)** | **Total (*N* = 419)** |
| --- | --- | --- | --- |
| Age *M*(*SD*) | 24.41 (5.94) | 21.26 (5.68) | 23.05 (6.02) |
| Age range | 16–50 years | 16–48 years | 16–50 years |
| Sex (n) | f = 77, m = 162 | f = 62, m = 118 | f = 139, m = 280 |
| ***Malaysian states (n)*** |  |  |  |
| Johor | 28 | 34 | 62 |
| Kedah | 11 | 6 | 17 |
| Kelantan | 14 | 2 | 16 |
| Kuala Lumpur | 22 | 23 | 45 |
| Labuan | 0 | 1 | 1 |
| Melaka | 9 | 5 | 14 |
| Negri Sembilan | 8 | 0 | 8 |
| Pahang | 13 | 8 | 21 |
| Penang | 8 | 16 | 24 |
| Perak | 16 | 20 | 36 |
| Perlis | 4 | 0 | 4 |
| Putrajaya | 1 | 0 | 1 |
| Sabah | 18 | 10 | 28 |
| Sarawak | 13 | 19 | 32 |
| Selangor | 65 | 29 | 94 |
| Trengganu | 9 | 5 | 14 |
| Not specified | 0 | 1 | 1 |
| No information provided | 0 | 1 | 1 |
| ***Religion (n)*** |  |  |  |
| Islam | 220 | 1 | 221 |
| Christianity | 16 | 34 | 50 |
| Buddism | 0 | 118 | 118 |
| Hinduism | 1 | 1 | 2 |
| Athiest | 2 | 19 | 21 |
| Taosim | 0 | 7 | 7 |
| ***Ethical affiliation (n)*** |  |  |  |
| Malay | 196 | 0 | 196 |
| Chinese | 4 | 162 | 166 |
| Indian | 1 | 0 | 1 |
| Iban | 6 | 0 | 6 |
| Bidayuh | 0 | 0 | 0 |
| Other Bumiputra Ethnic Groups | 29 | 18 | 47 |
| Eurasian | 0 | 0 | 0 |
| Other | 3 | 0 | 3 |
| ***Educational level (n)*** |  |  |  |
| Primary | 0 | 1 | 1 |
| Secondary | 72 | 94 | 166 |
| Tertiary | 132 | 80 | 212 |
| Professional | 35 | 5 | 40 |

*Note*. f = female, m = male, *M* = mean, *SD* = standard deviation, *n* = number.
