# Supplementary material for: Irrational beliefs in Bahasa Malaysia and Mandarin speaking populations: the cross-cultural validation of the irrational performance beliefs inventory
Source: BMC Psychol. 2025 Dec 1;13:1349. doi: 10.1186/s40359-025-03579-y (PMC12690909; doi:10.1186/s40359-025-03579-y)
Supplement: Supplementary file 2 — Supplementary Material 2 [file 40359_2025_3579_MOESM2_ESM.docx]

**Table S2 | 23-item iPBI-Mandarin**

| **Item**  **Nr.** | **Item** | **Dimension** |
| --- | --- | --- |
| 9 | 我需要别人认为我做出了有价值的贡献.  *I need others to think that I make a valuable contribution.* | PIB |
| 11 | 你是否曾經需要思考最明顯不過的事情（譬如自己的思維，每天都會執行的動作或每天都會接觸的物件.  *I absolutely should not be snubbed by people that matter to me.* | PIB |
| 13 | 我一定不可以被我的同伴们排挤.  *I must not be dismissed by my peers.* | PIB |
| 18 | 我需要被我的队员尊重.  *I have to be respected by the members of my team.* | PIB |
| 1 | 我不能忍受自己没有被给予机会.  *I can’t bear not being given chances.* | LFT |
| 3 | 我不能忍受没能达到我的目标这件事.  *I can’t stand not reaching my goals.* | LFT |
| 15 | 我不能忍受我在一件我很在意的事情上失败了.  *I can’t tolerate it when I fail at something that means a great deal to me.* | LFT |
| 20 | 我不能忍受在我所做的事情上没有取得进步.  *I can’t bear not getting better at what I do.* | LFT |
| 25 | 我不能接受我的能力没有持续地发展和进步.  *I couldn’t stand it if my competencies did not continually develop and improve.* | LFT |
| 6 | 没有被我的伙伴们公平对待会是件痛苦的事.  *It’s awful to not be treated fairly by my peers.* | AWF |
| 16 | 如果别人不认可我，这是很糟糕的.  *It’s awful if others do not approve of me.* | AWF |
| 17 | 如果别人认为我没有做出有用的贡献，这是件可怕的事.  *It’s awful if others think I do not make a valuable contribution.* | AWF |
| 21 | 被我的同伴们排挤是件可怕的事.  *It would be terrible to be dismissed by my peers.* | AWF |
| 23 | 如果别人不给我机会，那是一件很打击我的事.  *It is appalling if others do not give me chances.* | AWF |
| 26 | 如果我在队伍中的位置并不被保障，那是可怕的事.  *It would be awful if my position in my team was not secure.* | AWF |
| 28 | 如果队伍中的其他成员不尊重我，这会是件糟糕的事.  *It’s terrible if the members of my team do not respect me.* | AWF |
| 2 | 如果有人做出影响到我但不合理的的决定，那表示我是一个没有价值的人.  *If decisions that affect me are not justified, it shows that I am worthless.* | DEP |
| 7 | 如果别人认为我在我做的事情上表现不好，这代表我是没有价值的.  *If others think I am no good at what I do, it shows I am worthless.* | DEP |
| 8 | 如果我面对挫折，这显示了我有多愚蠢.  *If I face setbacks it goes to show how stupid I am.* | DEP |
| 10 | 如果我没有被给予机会，那表示我不是一个有价值的人.  *If I am not given opportunities, then it shows that I am not a worthwhile person.* | DEP |
| 14 | 如果我没有在对我来说重要的事情上取得成功，我就是一个失败者.  *I am a loser if I do not succeed in things that matter to me.* | DEP |
| 24 | 如果我在队伍中的立足之处并不稳当，那显示我是个没价值的人.  *If my position in my team was not secure, then it would show I am worthless.* | DEP |
| 27 | 如果我的能力没有持续发展和进步，这显示着我是个失败者.  *If my competencies did not continually develop and improve, it would show what a failure I am.* | DEP |

*Note.* Response format is from 1 (*strongly disagree*) to 5 (*strongly agree*). PIB = Primary irrational beliefs, LFT = Low frustration tolerance, AWF = Awfulization, DEP = Depreciation*. Removed items*: Item 4 (PIB): 我需要我的教练／经理人以尊重的方式对待我 (*Decisions that affect me must be justified*.), Item 5 (PIB): 我需要对我来说重要的人给予我好评 (*I have to be viewed favorably by people that matter to me.*), Item 22 (PIB): 影响到我的决定，必须是合乎道理的 (*I need my manager/coach to act respectfully towards me.*), Item 12 (LFT): 我不能忍受在我所做的事情上没有取得进步 (*I can’t bear not succeeding in things that are important to me.*), and Item 19 (LFT): 我不能接受我的能力没有持续地发展和进步 (*I can’t stand failing in things that are important to me.*).
