# Supplementary material for: Irrational beliefs in Bahasa Malaysia and Mandarin speaking populations: the cross-cultural validation of the irrational performance beliefs inventory
Source: BMC Psychol. 2025 Dec 1;13:1349. doi: 10.1186/s40359-025-03579-y (PMC12690909; doi:10.1186/s40359-025-03579-y)
Supplement: Supplementary file 3 — Supplementary Material 3 [file 40359_2025_3579_MOESM3_ESM.docx]

**Table S3 | 25-item iPBI-Malay**

| **Item**  **Nr.** | **Item** | **Dimension** |
| --- | --- | --- |
| 4 | Perlu dipertimbangkan keputusan yang melibatkan saya.  *Decisions that affect me must be justified.* | PIB |
| 5 | Saya perlu disenangi oleh orang yang penting kepada saya.  *I have to be viewed favourably by people that matter to me.* | PIB |
| 9 | Saya suka jika orang lain menghargai sumbangan saya.  *I need others to think that I make a valuable contribution.* | PIB |
| 11 | Saya tidak suka dihina oleh orang yang penting kepada saya.  *I absolutely should not be snubbed by people that matter to me.* | PIB |
| 13 | *Saya tidak ingin rakan-rakan sejawat memecatkan saya.*  *I must not be dismissed by my peers.* | PIB |
| 18 | Saya ingin dihormati oleh ahli-ahli pasukan saya.  *I have to be respected by the members of my team.* | PIB |
| 3 | Saya gelisah jika tidak mencapai matlamat.  *I can’t stand not reaching my goals.* | LFT |
| 12 | Saya tidak sanggup gagal dalam perkara-perkara yang penting kepada saya.  *I can’t bear not succeeding in things that are important to me.* | LFT |
| 15 | Saya tidak boleh menerima kegagalan saya dalam sesuatu yang amat bermakna kepada saya.  *I can’t tolerate it when I fail at something that means a great deal to me.* | LFT |
| 19 | Saya tidak boleh menerima kegagalan saya dalam sesuatu yang amat bermakna kepada saya.  *I can’t stand failing in things that are important to me.* | LFT |
| 20 | Saya rasa kecewa jika saya tidak bertambah baik pada apa yang saya lakukan.  *I can’t bear not getting better at what I do.* | LFT |
| 25 | Saya rasa kecewa jika saya tidak bertambah baik pada apa yang saya lakukan.  *I couldn’t stand it if my competencies did not continually develop and improve.* | LFT |
| 6 | Adalah sangat pahit jika tidak diberi layanan adil oleh rakan-rakan sejawat saya.  *It’s awful to not be treated fairly by my peers.* | AWF |
| 16 | Amat menyedihkan jika orang lain tidak menyukai saya.  *It’s awful if others do not approve of me.* | AWF |
| 17 | Amat terseksa jika orang lain menganggap saya tidak menyumbangkan sesuatu yang berharga.  *It’s awful if others think I do not make a valuable contribution.* | AWF |
| 21 | Adalah sangat pahit jika tidak diberi layanan adil oleh rakan-rakan sejawat saya.  *It would be terrible to be dismissed by my peers.* | AWF |
| 23 | Saya berasa resah jika tidak diberikan peluang.  *It is appalling if others do not give me chances.* | AWF |
| 26 | Amat terseksa jika kedudukan saya dalam pasukan tidak dijamin.  *It would be awful if my position in my team was not secure.* | AWF |
| 28 | Sangat mengecewakan jika ahli-ahli pasukan saya tidak menghormati saya.  *It’s terrible if the members of my team do not respect me.* | AWF |
| 7 | Saya rasa tidak bernilai, jika orang lain menganggap saya tidak cekap dalam bidang saya.  *If others think I am no good at what I do, it shows I am worthless.* | DEP |
| 8 | Jika saya menghadapi halangan, ia mencerminkan kebodohan saya.  *If I face setbacks it goes to show how stupid I am.* | DEP |
| 10 | Jika saya tidak diberi peluang, saya merasakan diri saya tidak berfaedah.  *If I am not given opportunities, then it shows that I am not a worthwhile person.* | DEP |
| 14 | *Saya rasa gagal sekiranya tidak berjaya dalam perkara-perkara yang penting bagi saya.*  *I am a loser if I do not succeed in things that matter to me.* | DEP |
| 24 | Jika tiada posisi untuk saya dalam pasukan, saya rasa diri saya tidak bernilai.  *If my position in my team was not secure, then it would show I am worthless.* | DEP |
| 27 | Saya rasa gagal jika tidak dapat mengembangkan atau meningkatkan kecekapan saya.  *If my competencies did not continually develop and improve, it would show what a failure I am.* | DEP |

*Note.* Response format is from 1 (*strongly disagree*) to 5 (*strongly agree*). PIB = Primary irrational beliefs, LFT = Low frustration tolerance, AWF = Awfulization, DEP = Depreciation. *Removed items*: Item 22 (PIB): Perlu dipertimbangkan keputusan yang melibatkan saya (*I need my manager/coach to act respectfully towards me*), Item 1 (LFT): Saya gelisah jika tidak mencapai matlamat. (*I can’t bear not being given chances*) and Item 2 (DEP): Jika saya menghadapi halangan, ia mencerminkan kebodohan say (If decisions that affect me are not justified, it shows that I am worthless).
